# Cellular Concentration of Survivin and Caspase 3 in Habitual Tobacco Chewers with and without Oral Squamous Cell Carcinoma in South Indian Rural Population—A Case Control Study

**DOI:** 10.3390/diagnostics12092249

**Published:** 2022-09-18

**Authors:** Susanna Theophilus Yesupatham, C. D. Dayanand, S. M. Azeem Mohiyuddin

**Affiliations:** 1 Department of Biochemistry, Sri Devaraj Urs Academy of Higher Education and Research, Tamaka, Kolar 563103, Karnataka, India; 2 Allied Health and Basic Sciences, Sri Devaraj Urs Academy of Higher Education and Research, Tamaka, Kolar 563103, Karnataka, India; 3 Department of Otorhinolaryngology and Head and Neck Surgery, Sri Devaraj Urs Academy of Higher Education and Research, Tamaka, Kolar 563103, Karnataka, India

**Keywords:** Survivin, Caspase 3, buccal cells, oral squamous cell carcinoma, tobacco chewers

## Abstract

Background: There is paucity of data on tissue levels of Survivin and Caspase 3 in south Indian tobacco chewers with oral Squamous cell carcinoma (OSCC). Oral cancer is a rapidly growing, highly prevalent head and neck malignancy; it involves a mucosal epithelium of a buccal cavity exposed to tobacco and other carcinogens. The basis of the survival of a tumor cell or transformed normal cell into a neoplastic cell is by the suppression of apoptosis regulation. Recently, researchers have focused on Survivin, an inhibitor of apoptosis family of proteins (IAP), involved in apoptosis regulation in cancer cells targeting the executioner Caspase 3. The current study aims to quantify the cellular levels of Survivin and Caspase 3 in tobacco chewers with OSCC and in habitual tobacco chewers without OSCC, in comparison to controls. Methods: A single centric case control study included 186 study subjects, categorized into: Group I (*n* = 63), habitual tobacco chewers with OSCC; Group 2 (*n* = 63), habitual tobacco chewers without OSCC; and Group 3 (*n* = 63), the controls. Resected tumor tissue from Group 1 and buccal cell samples from Groups 2 and 3 were collected into phosphate buffer saline (PBS) and assayed for Survivin and Caspase 3 levels by the ELISA sandwich method. Results: The mean ± SD of the Survivin protein in Group 1 was (1670.9 ± 796.21 pg/mL); in Group 2, it was (1096.02 ± 346.17 pg/mL); and in Group 3, it was (397.5 ± 96.1 pg/mL) with a significance of *p* < 0.001. Similarly, the level of Caspase 3 in Group 1 was (7.48 ± 2.67 ng/mL); in Group 2, it was (8.85 ± 2.41 ng/mL); and in Group 3, it was (2.27 ± 2.24 ng/mL) with a significance of *p* < 0.001. Conclusion: The progressive transformation of buccal cells to neoplastic cells is evident; in the case of OSCC, this indicates that the over-expression of Survivin compared to Caspase 3 confirms the suppression and dysregulation of apoptosis.

## 1. Introduction

In India, head and neck cancers constitute 30–35% of all the cancers; in addition, the majority of them are oral cancers. Head and neck cancers are the sixth most common malignancy, accounting for 2–4% of cancer cases globally. Oral cancer comprises a group of neoplasm seen in any region of the oral cavity. In total, 90% of the oral malignancies are OSCC; they occur most frequently on the oral mucosal epithelium [1,2,3,4]. According to hospital-based statistics of the study area, oral cancer is found to be the most common cancer observed in both sexes; it has a record of around 30% [5].

Mortality rate is high in oral cancer patients and the survival rate in the advanced disease of oral squamous cell carcinoma is hardly 40% to 50%. Patients with oral cancer generally present at late stages of cancer when symptoms of an oral or neck mass appear; and there is pain and bleeding due to the tumor. Previous studies have observed that the 5-year survival rate is 85%, provided that the oral cancer could be identified in the early stages; this emphasizes the vital importance of early detection [6].

OSCC sets by molecular mechanisms induced by multiple molecules either from chemical carcinogens; tobacco; alcohol; betel quid; and biological factors, such as human papillomavirus (HPV), syphilis, oro-dental factors and dietary deficiencies [7].

Among the various risk factors predisposing to oral cancer, the primary risk factors include tobacco and alcohol consumption [8,9]. The International Agency for research on Cancer (IARC) classifies tobacco as a group I carcinogenic substance in the oral cavity [10]. There is much evidence that suggests the human papilloma virus (HPV16) as a risk factor for oropharyngeal cancer [10,11,12]. The reported profile in India is not consistent with the global statistics reporting the incidence of HPV in oral cancer to range from 0 to 75% [11,12,13,14]. However, a study done among the local population with OSCC has shown none of the tumor specimens to be HPV positive [15].

Histopathological examination of a biopsy specimen from the suspicious lesion is the gold standard for the diagnosis of oral cancer. However, the procedure is time-consuming and the histopathology report will be reliable only when the specimen is representative due to field cancerization and condemned mucosa. The procedure is quite unpleasant for the patient and will not be suitable for community screening of the high-risk population for early diagnosis during the asymptomatic phase of the disease [3].

Early identification remains the key element for effective treatment strategies. Survivin, the smallest member, as an inhibitor, of the apoptosis protein (IAP) family, is distinct from other IAPs by virtue of its expression seen in embryonic and fetal tissues and in tumor cells; however, it has undetectable or low expression in normal adult differentiated tissues. The exact mechanism by which Survivin inhibits apoptosis is still unknown; various reports suggest that Survivin inhibits apoptosis by directly or indirectly interacting with executioner Caspase 3, 6 and 7 and disrupting the caspase cascade to prevent apoptosis of the tumor cells [16,17,18].

Over-expression of Survivin is reported in various human malignancies, such as in breast (90.2%), liver (87%), ovary (73.5%), bladder (57.8%), lung (85.5%), stomach (68%), esophageal (80%), oral (>75%) and hematological malignancies (68%) [19]. Research reports mentioned that Survivin expression induced transcriptional changes in the tissue microenvironment, promoting tumorogenesis in the bladder tissue [20]. The above studies were based on predominantly immuno-histochemistry and mRNA expression. Literature mentioned higher Survivin expression in an oral squamous cell carcinoma by an immunohistochemistry study; and in another study, reported higher expression of Survivin as a critical factor for radio resistance in head and neck squamous cell carcinoma (HNSCC) cell lines [21,22]. However, the quantitative levels of Survivin and Caspase 3 in an oral squamous cell carcinoma resulting from tobacco chewing has not been studied so far. Therefore, we decided to estimate the tissue levels of Survivin and Caspase 3 in tobacco chewers with OSCC; and to evaluate the association of the same with buccal cell levels in habitual tobacco chewers without OSCC and in the controls.

## 2. Materials and Methods

### 2.1. Patients

Study Design: a single centric case control study carried out from 2019 to 2022; the study was approved by the central ethics committee in a vide no. SDUAHER/KLR/CEC/33/2018-19, dated 14 May 2018. The study population included 186 participants with age and gender matched, enrolled from the Dept. of Otorhinolaryngology and Head and Neck Surgery, R.L. Jalappa Hospital and Research Center; this is a rural tertiary care hospital attached to the Sri Devaraj Urs Medical College, Tamaka, Kolar, Karnataka, India. Individuals aged between 30–65 years were included in the study. The study participants were categorized into three groups: Group 1 (*n* = 63), tobacco chewers with OSCC, clinically obvious and histopathological confirmed cases; Group 2 (*n* = 63), habitual tobacco chewers without any precancerous or cancerous lesion in the oral cavity; and Group 3 (*n* = 63), non-tobacco chewers as healthy controls. A detailed clinical history; and a local and systemic examination of the participants were recorded in a structured proforma.

The study excluded patients with recurrent or chronic ulcerative lesions of the oral cavity, such as pemphigus/Behcet’s syndrome; patients who had undergone radiotherapy, previous oncosurgery or orneoadjuvant chemotherapy in the past; and patients with immunodeficiency.

### 2.2. Methods

#### 2.2.1. Sample Collection

Resected tissue specimens of primary OSCC of buccal mucosa were collected in a phosphate buffered saline PBS (pH 7.2–7.4). Tissue homogenate was prepared from the tissue specimen. Buccal cell scrapings were obtained from the Group 2 and Group 3 subjects; the participants were asked to rinse their mouth thoroughly with distilled water; and soft buccal brushes obtained from the pure gene buccal cell kit from Qiagen, Maryland, USA were used for the buccal cell sample collection. The buccal brushes were twirled and brushed gently for 15 secs along the left inner and right inner cheeks; upper and lower gingivo buccalsulci; and the anterior aspect of the buccal mucosa. The buccal brushes, after scraping, were suspended in 1.0 mL of PBS containing vials and preserved at −20 °C [23]. The buccal cell viability and count was determined using the stain trypan blue; and counted using a haemocytometer.

#### 2.2.2. Buccal Tissue Sample Analysis

The tissue specimens were processed as per the kit manufacturer instructions. In total, 500 mg of tissue specimen was minced and placed in a vial containing PBS and a radio-immunoprecipitation assay (RIPA) lysis buffer; this was vortexed and incubated at room temperature for 30–45 min, the tissue homogenate was centrifuged at 5000× *g* for 5 mins, and the supernatant was separated and stored at −20 °C until analysis.

The buccal cell samples were centrifuged for 20 min at 3000 rpm; the supernatant was discarded. The cell suspension was diluted with PBS and RIPA buffer; vortexed and incubated for 30 min; centrifuged at 5000× *g* for 5 min; and the supernatant was stored at −20 °C until analysis.

#### 2.2.3. Human Survivin Assay

The supernatant obtained from the above steps was used for quantification of human Survivin by the sandwich ELISA method (K12-5528, kinesis Dx, Los Angeles, CA, USA). Samples and standards were added to the monoclonal antibody precoated microwells; the human Survivin present in the standard and sample was bound by antibodies; and the biotin-labeled antibody was added, followed by the addition of a streptavidin horse radish peroxidase (HRP) and incubated to form a complex. The wells were washed and a 3,3′,5,5′Tetramethylbenzidine (TMB) substrate was added. The color developed is proportional to the amount of human Survivin in the sample. The absorbance of yellow color developed was measured at 450 nm. The standards were analyzed in duplicate for the preparation of a standard curve. The assay range of human Survivin is 31.259 pg/mL to 200 pg/mL.

#### 2.2.4. Human Cysteinyl Aspartate Specific Proteinase 3 (Caspase 3) Assay

The supernatant obtained from the above steps was used for the quantification of human cysteine aspartate specific proteinase −3 by the sandwich ELISA method as per the kit manufacturer instructions (K12-0970 kinesis Dx, Los Angeles, CA, USA). Samples and standards were added to the monoclonal antibody precoated microwells; the human Caspase 3 present in the standard and sample was bound by antibodies; and the biotin-labeled antibody was added, followed by the addition of streptavidin HRP and incubated to form a complex. The wells were washed and a TMB substrate was added. The color developed is proportional to the amount of human Caspase 3 in the sample. The absorbance of yellow color developed was measured at 450 nm. The standards were analyzed in duplicate for the preparation of a standard curve. The assay range for Caspase 3 is 0.312 ng/mL–20 ng/mL.

### 2.3. Statistical Analysis

The data obtained from the study groups were statistically analyzed using licensed version SPSS Software version 22 (IBM USA). The Shapiro–Wilk test was carried out to evaluate the normality of the data. The continuous data are represented as mean and standard deviation. An independent student t test was used to compare the expressed levels of Survivin and Caspase 3 in the buccal cells and tumor tissues. The one way analysis of variance (ANOVA) test was used as a test of significance to identify the mean difference to compare the continuous variables (*p* value). Pearson’s correlation was used to describe the correlation between the Caspase 3 and Survivin levels within the groups (r value). The statistical significance was considered with a *p* value < 0.05.

## 3. Results

### 3.1. Patients Characteristic Details

The participants from the study area belong to poor nutritional and lower socio-economic status. The majority of the oral cancer patients that visited the hospital for treatment had advanced disease, tumor node metastasis. The staging of the OSCC patients in the study was based on the American Joint Committee on cancer staging manual: eighteen patients (28.6%) were T4N1; eleven patients (17.5%) were T3N; five patients (7.9%) were each T2N0, T2N1 and T3N0, respectively; four patients (6.3%) were T4N0; and three patients (4.8%) were each T2N2b, T3N2b and T4N2a, respectively. All the patients with oral cancer had a Grade 1 well-differentiated Squamous cell carcinoma.

### 3.2. Survivin and Caspase 3 Analysis

**Figure 1:**
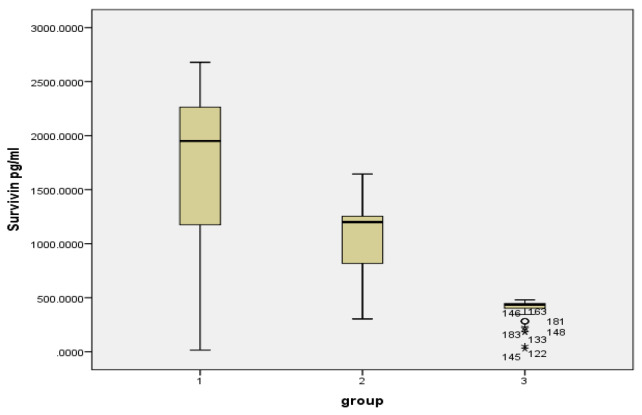
Survivin levels (pg/mL) in the study groups. Group 1: Tobacco chewers with OSCC, Group 2: Habitual tobacco chewers without any precancerous or cancerous lesion in the oral cavity, Group 3: Controls.

**Figure 2:**
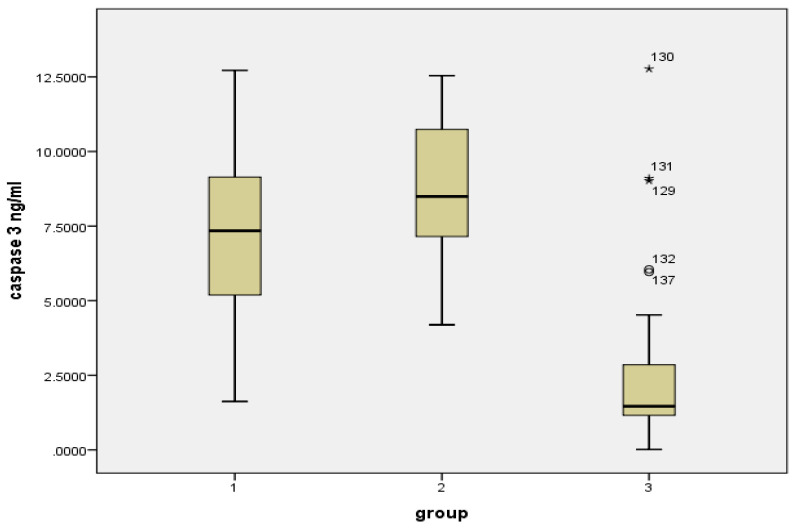
Caspase 3 levels (ng/mL) in the study groups. Group 1: Tobacco chewers with OSCC, Group 2: Habitual tobacco chewers without any precancerous or cancerous lesion in the oral cavity, Group 3: Controls.

**Figure 3:**
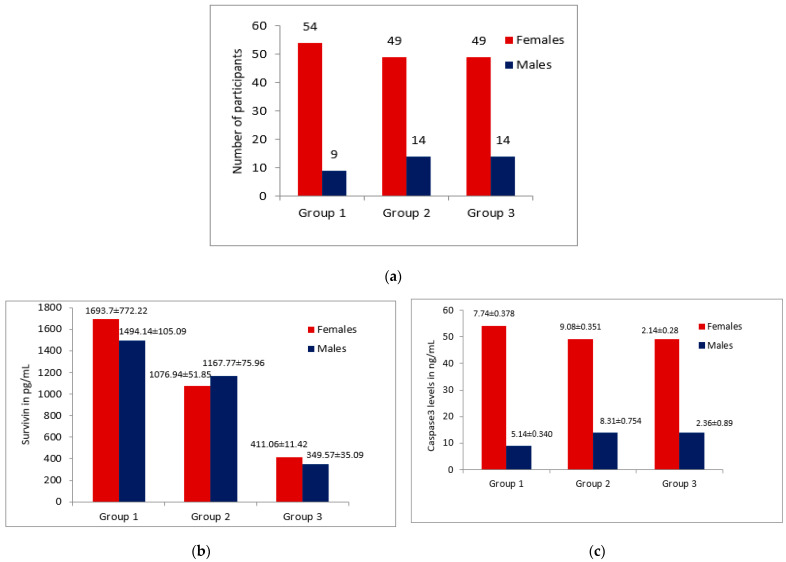
(**a**) shows the number of gender-wise participants in each group; (**b**) shows the mean ± std error of the mean of Survivin in pg/mL among the females and males in each group; and (**c**) shows the mean ± std error of the mean of the Caspase 3 levels in ng/mL among the females and males in each group.

The study showed a statistically significant elevation of tissue Survivin levels in the OSCC patients (1670.9 ± 796.21 pg/mL) compared to the levels in buccal cell samples of habitual tobacco chewers (1096.02 ± 346.17 pg/mL) and healthy controls (397.5 ± 96.1 pg/mL) with a *p* value < 0.001. A statistically significant reduction was observed in the Caspase 3 tissue levels in the OSCC patients (7.48 ± 2.67 ng/mL) compared to the buccal cell samples of habitual tobacco chewers (8.85 ± 2.41 ng/mL) and the controls (2.27 ± 2.24 ng/mL) with a *p* value < 0.001; this is shown in Table 1, Figure 1 and Figure 2. However, there was no significant variation in the levels of Survivin and Caspase 3 in the female and male patients with OSCC or in the habitual tobacco chewers group; this is shown in Figure 3.

## 4. Discussion

The present research was carried out to assess the influence of tobacco consumption on the levels of the anti-apoptotic protein Survivin and Caspase 3 concentration in the study participants. To the best of our knowledge, as per the available literature, the current study is the first to quantify Survivin and Caspase 3 concentrations in tissue samples and buccal cell scrapings of OSCC patients, habitual tobacco chewers and in healthy controls without any tobacco habits.

Our research findings revealed that the majority of the individuals with oral cancer were female patients (%), with a male to female ratio of 1:7; in addition, most of the OSCC patients were in the age group of 45–65 years. The reason could be due to the fact that majority of the women in this region are addicted to tobacco chewing and tobacco quid consumption. However, the male subjects are more addicted to smoking and alcohol consumption. A possible explanation might be that people in this region become addicted to tobacco at a younger age, and many of them develop oral cancer much later. Moreover, illiteracy and poor economic conditions are also contributing factors to chewable tobacco addiction in this region. In support of our observation, a few research reports are also available in agreement with the results of this study [5,24,25,26].

In the year 2021, Survivin expression by Immunohistochemistry (IHC) in a pediatric Ewing sarcoma reported by A. M. Mahmoud and his coworkers demonstrated a significantly higher positive Survivin expression in males compared to female patients [27]. However, in the current study groups, there were no significant differences in the Survivin levels among the female and male patients in Group 1 and Group 2; however, in the healthy control Group 3, the mean Survivin levels are significantly higher among females compared to their male counterparts (*p* = 0.034). Our findings align with the Survivin IHC expression demonstrated by Angelin et al. (2020), and the salivary Survivin levels indicated by Santarelli et al. (2013) in OSCC; herein, the authors did not find any statistical significance in relation to gender and Survivin expression [21,28]. In order to determine the variation in Survivin levels with respect to gender, further studies with larger sample sizes are required.

Among the tobacco chewers with OSCC, the majority of the patients had advanced disease; with 18 patients in Stage III and 31 patients in Stage IV of the total 63 OSCC patients. This could be due to the lack of awareness among the high-risk groups regarding the early signs of premalignant and malignant lesions in the oral cavity. They tend to ignore the asymptomatic early lesions in the oral cavity; in addition, they commonly present with advanced disease when the symptoms of the lesions tend to appear [25,26].

In the present study, there was a significant elevation in tissue Survivin levels and significantly reduced tissue Caspase 3 levels in tobacco chewers with OSCC; as compared to tobacco chewers without OSCC and in the control group in Table 2. The findings of this study are in accordance with the observations on serum Survivin levels reported by S. X. Li et al. Among the Chinese patients with oral cancer, the authors also observed the high mRNA expression of Survivin in OSCC tissue; however, the expression of Caspase 3 was not detected in tumor tissue [29]. Yet another study by C. Jane et al. observed the increased expression of Survivin in OSCC by the IHC method [30]. Gunaldi, M. et al. studied the increased levels of serum Survivin levels in the colon, and ovarian and other cancer patients were compared to healthy subjects; they concluded that the high Survivin levels showed a four-fold increased risk for cancer in the subjects with a high suspicion for cancer [31].

Survivin has been investigated in various other cancers, such as pancreatic, adenocarcinoma, esophageal cancers, bladder and breast cancers; these studies have predominantly determined the mRNA expression levels in the cells, and found the expression levels of Survivin mRNA to be significantly elevated. This substantiates the key role of Survivin in the inhibition of apoptosis and malignant transformation of cells; further, the significant increase in the expression of Survivin in high-grade tumors hints at the structural and functional activity linked to apoptosis resistance [32,33,34].

The majority of the secondary data on Survivin detection were based on IHC or Western blot techniques; these are time-consuming and have constraints with obtaining samples for testing and screening among a high-risk population. For the first time, we investigated a quantitative measurement of Survivin and Caspase 3 levels using ELISA in tumor tissue extracts and buccal cell lysates in the OSCC group, habitual tobacco chewers and healthy controls. This is because this method has monoclonal antibodies raised to Survivin and Caspase 3; thereby, this assay procedure is simple, sensitive and specific to the analyte that might be used for the screening of a high-risk population.

**Figure 4:**
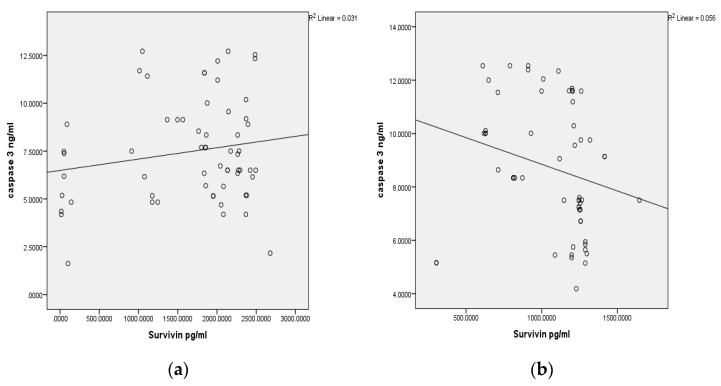

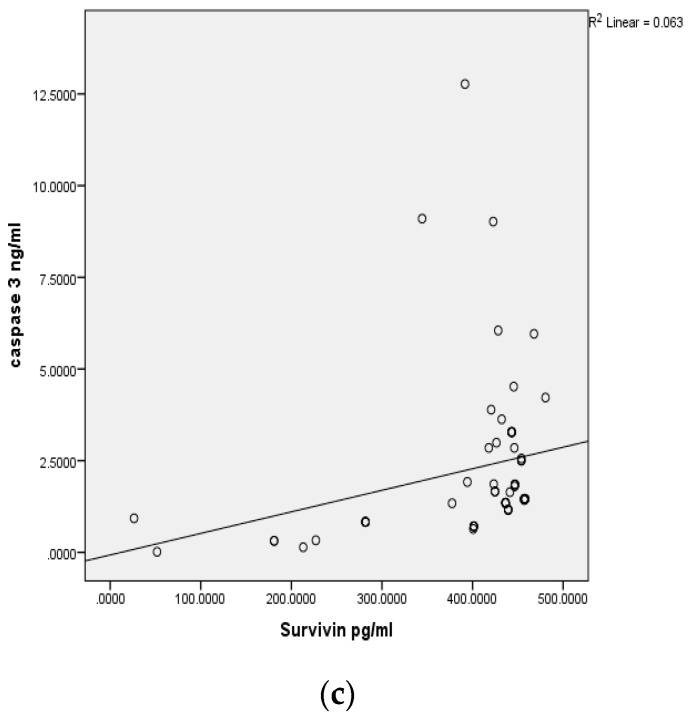
(**a**) Correlation analysis of the Survivin and Caspase 3 levels in tissue specimens of the OSCC patients; *p* = 0.174. (**b**) Correlation analysis of the Survivin and Caspase 3 levels in the buccal cell samples of the tobacco chewers; *p* = 0.646. (**c**) Correlation analysis of the Survivin and Caspase 3 levels in the buccal cell samples of the control subjects. There is a positive significant correlation between Caspase 3 and Survivin among the controls; *p* = 0.046.

Furthermore, in our study, there was a positive correlation (r = 0.031) between the Survivin and Caspase 3 levels in the tissue samples of the OSCC patients; and a negative correlation (r = 0.056) between the Caspase 3 and Survivin levels in the buccal cell samples of the tobacco chewers without OSCC. Nevertheless, the correlation was not statistically significant in Figure 4. This information shows that the expression of Survivin is independent of Caspase 3 levels in the tumor cells and buccal cells. The probable explanation could be that any genetic alteration is confined to the baculoviral IAP repeat containing five (BIRC5) gene codes for Survivin protein excessive synthesis or functional alteration; this is so in order to preserve the survival and proliferation of the transformed mucosal cells, which needs to be further explored.

Tobacco contains numerous carcinogens (nicotine) directly or indirectly causing tumorogenesis by virtue of their absorption in the oral epithelium. Predominantly, nicotine is reported to have a direct role in tumorogenesis as demonstrated by studies on cell culture and animal models. Nicotine and its adducts, through the Akt-dependent pathway, are known to reduce potential of chemotherapeutic drugs by up-regulating the expression of the apoptosis inhibitor Survivin as a key protein [35,36].

Though the mechanisms to resist programmed cell death by Survivin are complex in nature, it is largely witnessed in various studies that Survivin considerably contributes to the inhibition of apoptosis in cancer cells. Further, there are various reports that suggest the indirect and direct binding of Survivin to Caspases 3, 6 and 7; thereby, disrupting the caspase cascade and cleavage mediated by caspases, and resulting in reduced apoptosis [17,37].

In the present study, nine of the OSCC patients and fourteen participants of the habitual tobacco chewers had the habit of regular consumption of alcohol (ethanol). Ethanol is hypothesized to be metabolized by oral micro flora into acetaldehyde, which is a known carcinogenic substance; however, the role of alcohol predisposing to the risk of developing oral cancer is still unclear [38].

The present data obtained can be considered as preliminary observations. The differential expression of Survivin in OSCC and normal cells suggests that Survivin serves as a potential cancer therapy target; will facilitate tailoring therapeutic strategies; and can be chosen as a community noninvasive screening test (CNST) in habitual tobacco chewers, as well as for the prognosis of OSCC patients during management as buccal cell samples are easy to collect and patient-compliant.

## 5. Conclusions

The study results conclude that there is an increased concentration of Survivin in the OSCC tissues in comparison to the cells exposed to the toxic effects of tobacco and the normal buccal mucosal cells. Moreover, there are also decreased Caspase 3 levels in OSCC compared to the habitual tobacco chewers and the controls. The quantification of Survivin and Caspase 3 can serve as a basis for screening. Buccal cell scraping is an easy and a safe technique to determine Survivin and Caspase 3 for the purpose of community screening of OSCC among a high-risk population.

## 6. Limitations of the Study

As it is a single centric study, the enrolled cases were not followed up for the assessment of the prognostic role of Survivin; the paired comparison of tissue survivin and its IHC expression in tumor tissue was not carried out. Further, the genetic analysis of the Survivin gene to know the possible polymorphism in the study subjects, and to correlate with the expression levels and its molecular function in the OSCC onset and progression, are the limitations of this study.

## Figures and Tables

**Table 1:** Mean ± SD of the Survivin and Caspase 3 levels in the study groups.

Analytes	Groups	Mean ± SD	95% Confidence Interval for Mean		*p* Value
			Lower Bound	Upper Bound	
Survivin (pg/mL)	Group 1	1670.9 ± 796.21	1466.94	1874.75	<0.001
	Group 2	1096.02 ± 346.17	1008.11	1183.93	
	Group 3	397.5 ± 96.1	373.29	421.69	
Caspase 3 (ng/mL)	Group 1	7.48 ± 2.67	6.80	8.17	<0.001
	Group 2	8.85 ± 2.41	8.24	9.46	
	Group 3	2.27 ± 2.24	1.70	2.83	

**Table 2:** Shows the ANOVA to test the significance of difference in the variables across the groups.

Multiple Comparisons							
Dependent Variable	(I) Group	(J) Group	Mean Difference (I–J)	Std. Error	*p* Value	95% Confidence Interval	
						Lower Bound	Upper Bound
Caspase 3 (ng/mL)	1	2	−1.37 *	0.441	0.002 *	−2.24	−0.50
		3	5.22 *	0.440	0.001 *	4.35	6.08
	2	1	1.37 *	0.441	0.002 *	0.50	2.24
		3	6.59 *	0.438	0.001 *	5.72	7.45
	3	1	−5.22 *	0.440	0.001 *	−6.08	−4.35
		2	−6.59 *	0.438	0.001 *	−7.45	−5.72
Survivin (pg/mL)	1	2	574.83 *	90.34	0.001 *	396.60	753.07
		3	1273.37 *	89.96	0.001 *	1095.84	1450.90
	2	1	−574.83 *	90.34	0.001 *	−753.07	−396.60
		3	698.54 *	89.61	0.001 *	521.73	875.34
	3	1	−1273.37 *	89.98	0.001 *	−1450.90	−1095.84
		2	−698.54 *	89.61	0.001 *	−875.34	−521.73

* The mean difference is significant at *p* < 0.05.

## Data Availability

The data presented in this study are available on request from the corresponding author.
